# Supplementary figures and images for: Fuzhengjiedu San inhibits porcine reproductive and respiratory syndrome virus by activating the PI3K/AKT pathway
Source: PLoS One. 2024 May 6;19(5):e0283728. doi: 10.1371/journal.pone.0283728 (PMC11073700; doi:10.1371/journal.pone.0283728)

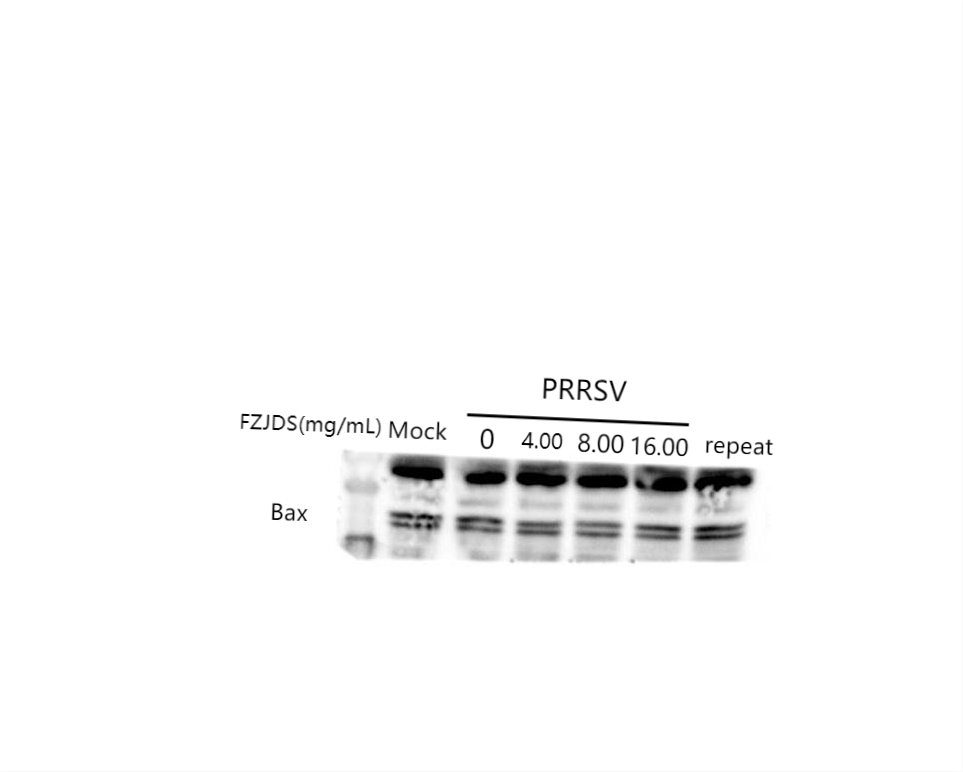

Supplement: S1 Data — (ZIP) [file pone.0283728.s001.zip › WB╘¡═╝/Bax/BAX.tif]

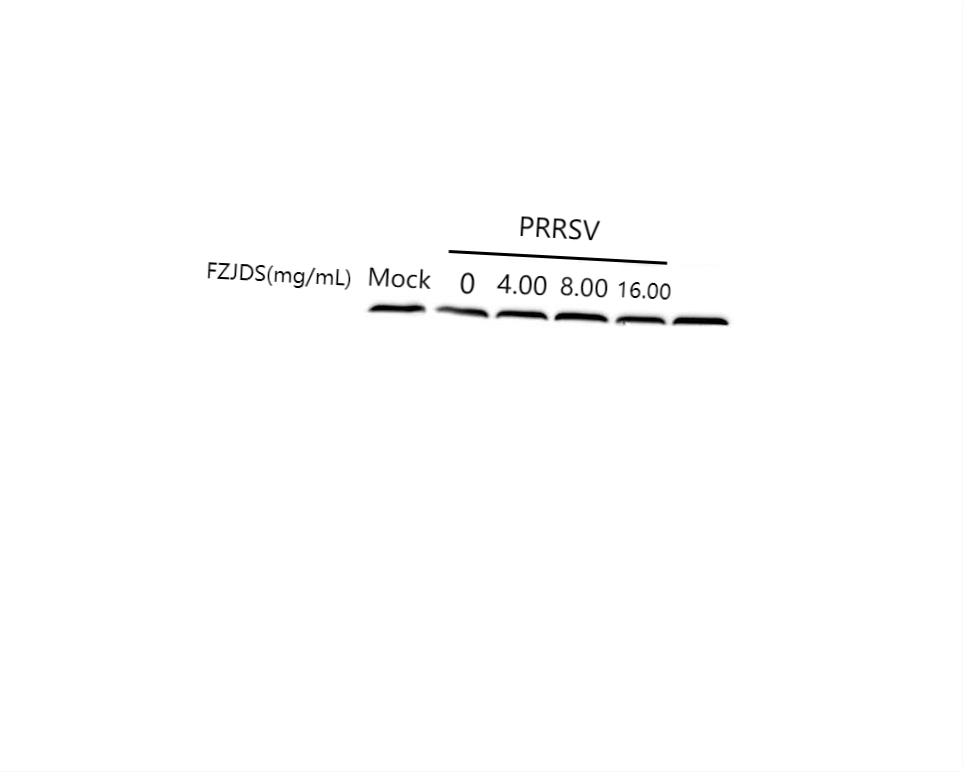

Supplement: S1 Data — (ZIP) [file pone.0283728.s001.zip › WB╘¡═╝/Bax/a┬-Actin.tif]

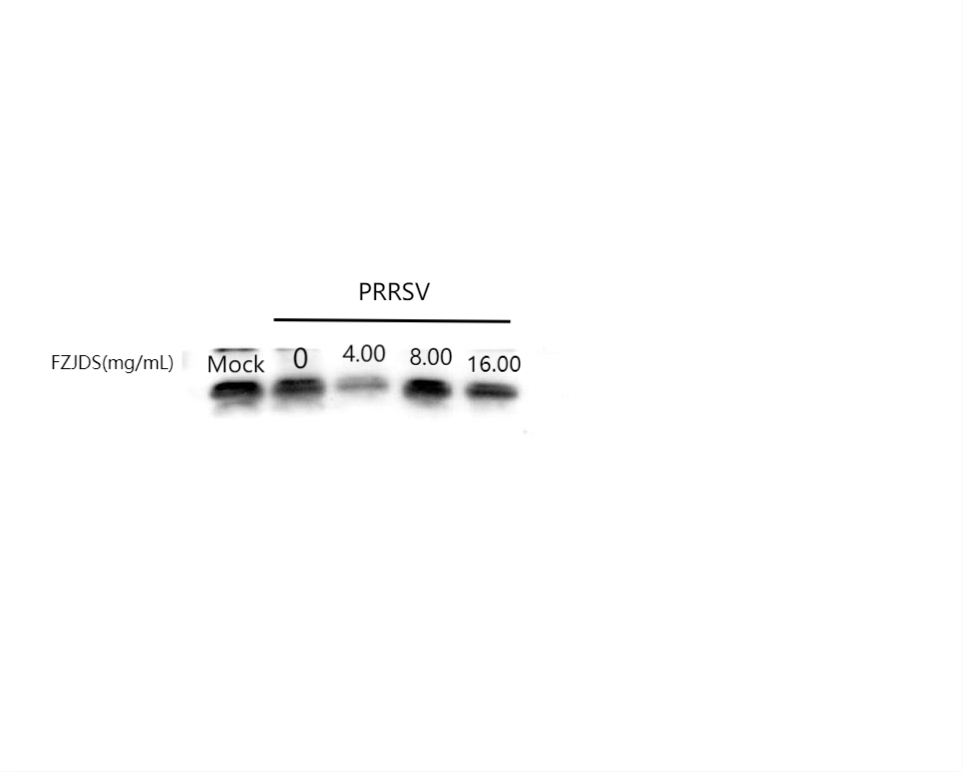

Supplement: S1 Data — (ZIP) [file pone.0283728.s001.zip › WB╘¡═╝/Bcl-2╥⌐╬∩╫Θ/bcl-2.tif]

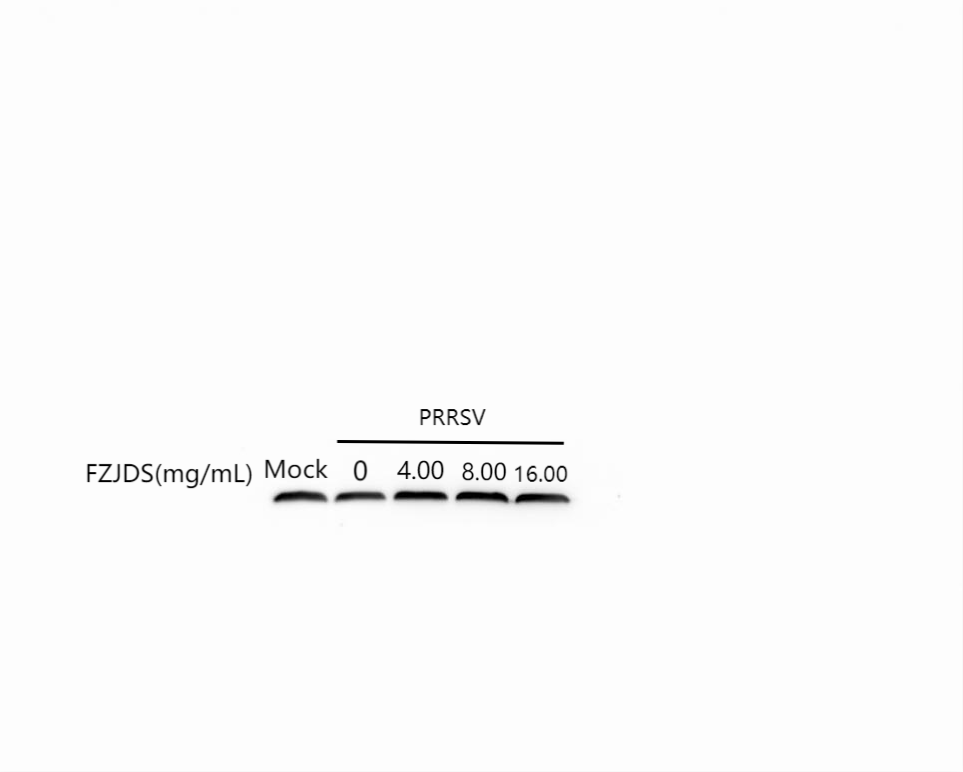

Supplement: S1 Data — (ZIP) [file pone.0283728.s001.zip › WB╘¡═╝/Bcl-2╥⌐╬∩╫Θ/a┬-Actin.tif]

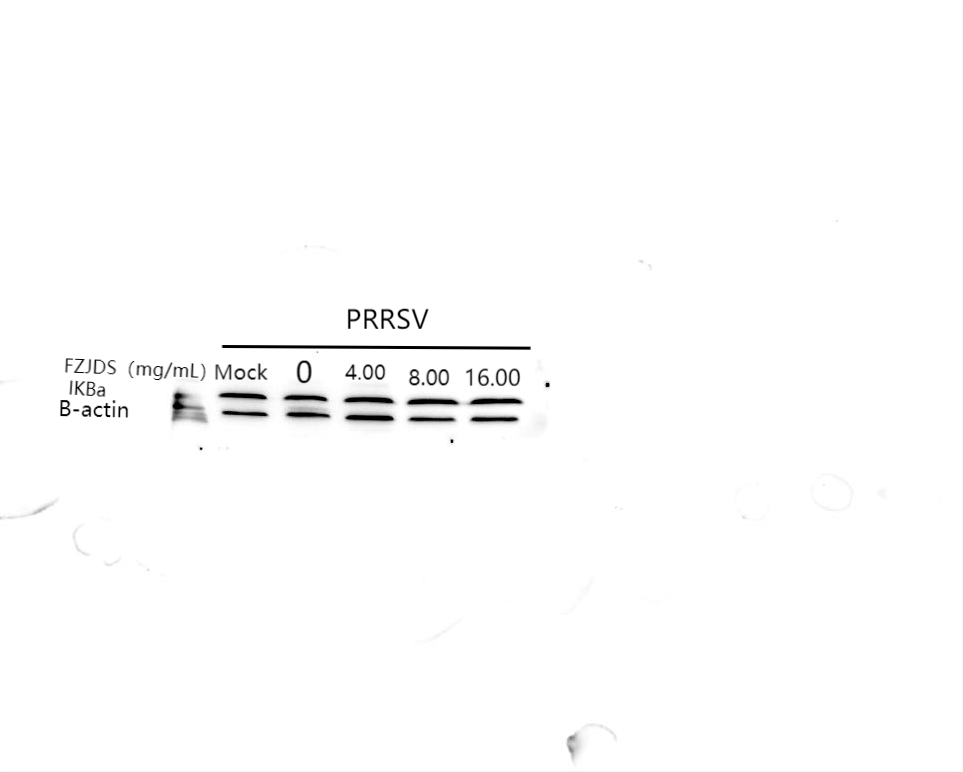

Supplement: S1 Data — (ZIP) [file pone.0283728.s001.zip › WB╘¡═╝/IKB/IKB+a┬-Actin.tif]

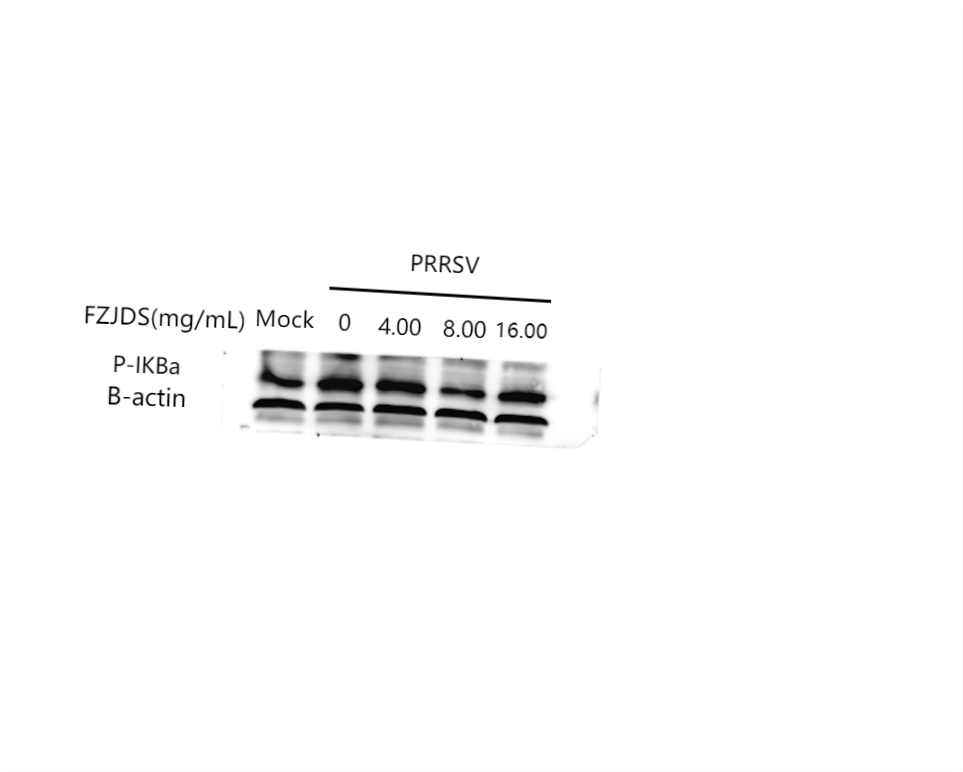

Supplement: S1 Data — (ZIP) [file pone.0283728.s001.zip › WB╘¡═╝/IKB/P-IKB+a┬-Actin.tif]

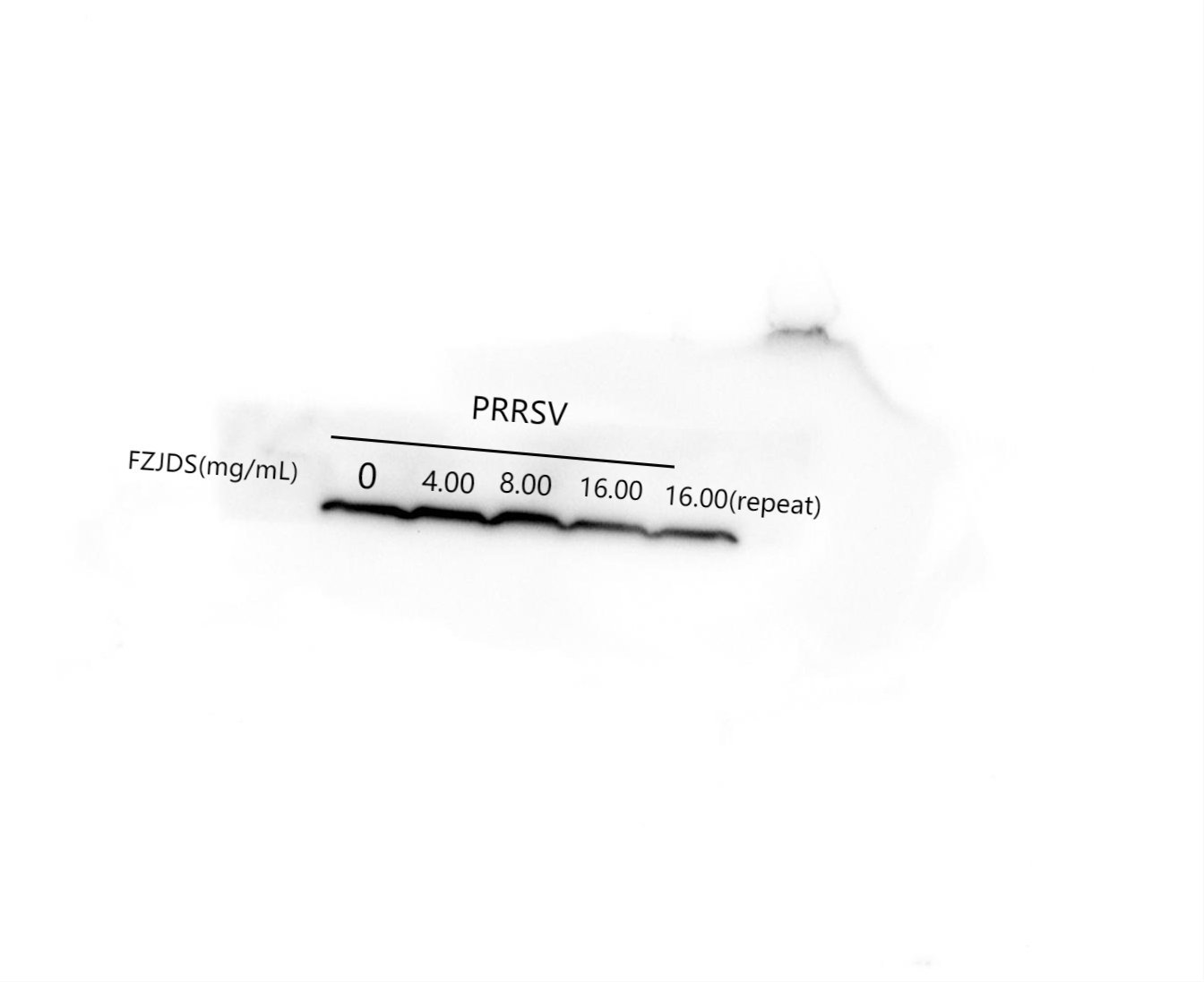

Supplement: S1 Data — (ZIP) [file pone.0283728.s001.zip › WB╘¡═╝/N╥⌐╬∩╝┴┴┐╫Θ/N╡░░╫.tif]

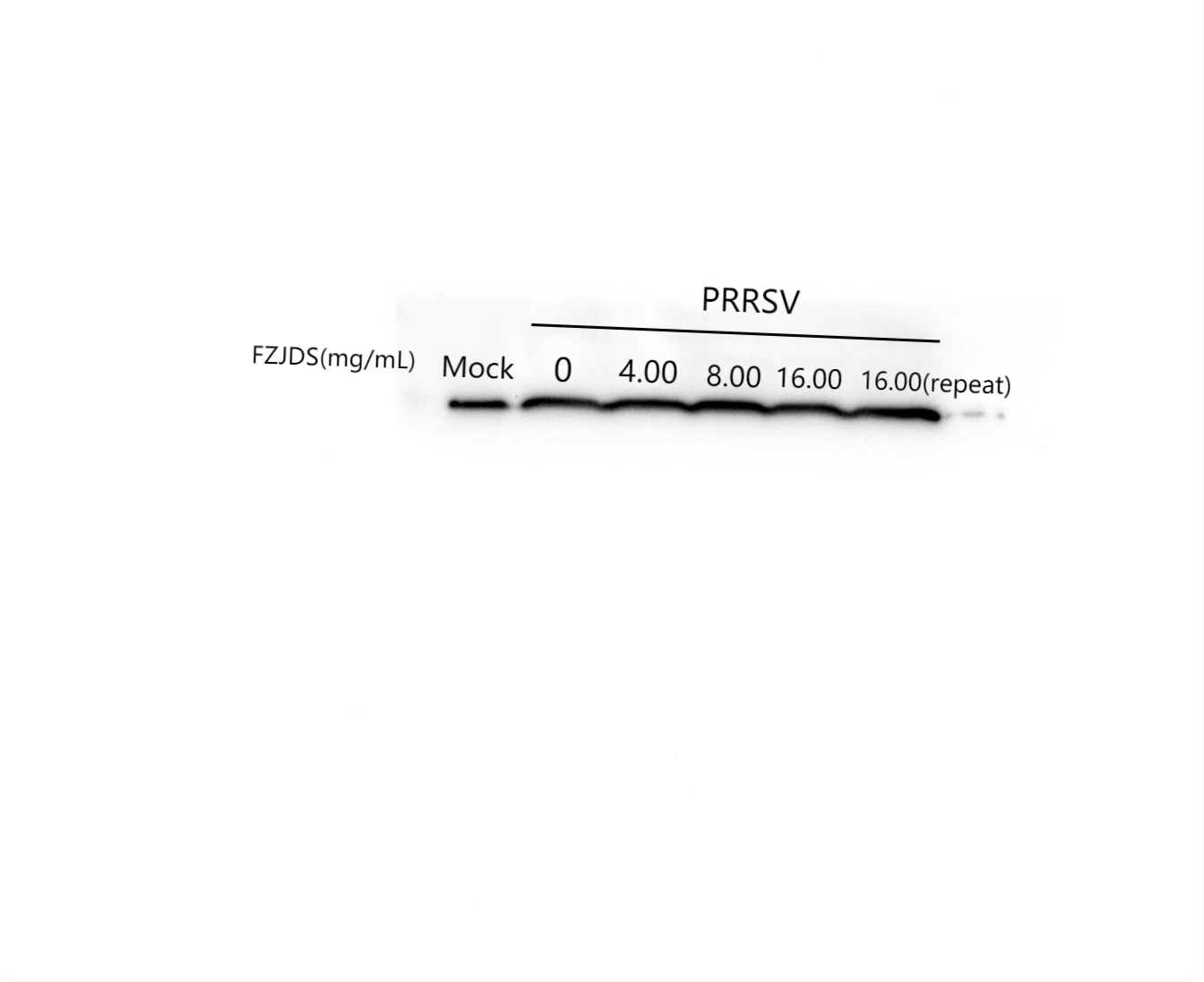

Supplement: S1 Data — (ZIP) [file pone.0283728.s001.zip › WB╘¡═╝/N╥⌐╬∩╝┴┴┐╫Θ/a┬-Actin.tif]

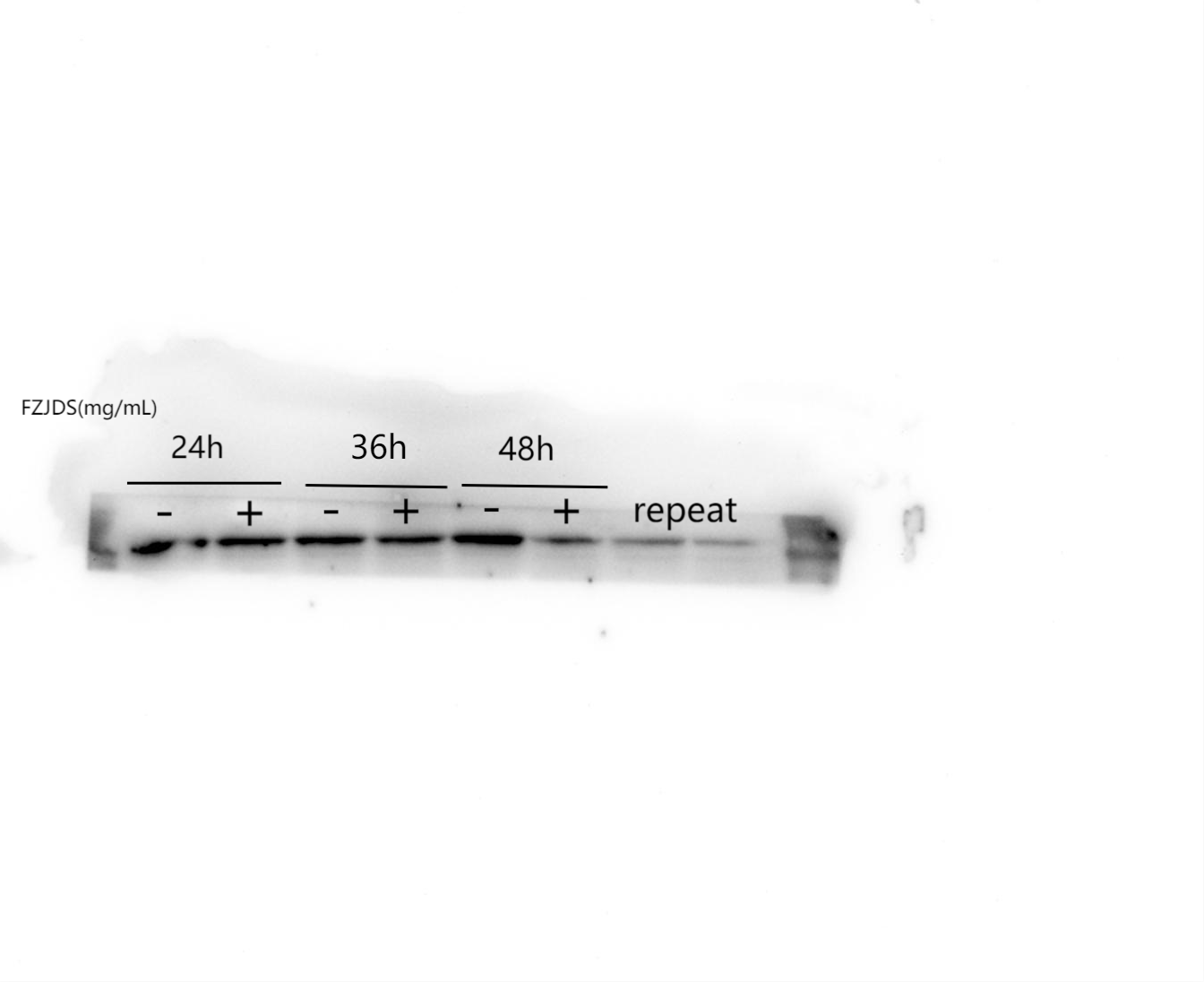

Supplement: S1 Data — (ZIP) [file pone.0283728.s001.zip › WB╘¡═╝/N╡░░╫╩▒╝Σ╥└└╡╫Θ/N╡░░╫╩▒╝Σ╥└└╡╫Θ.tif]

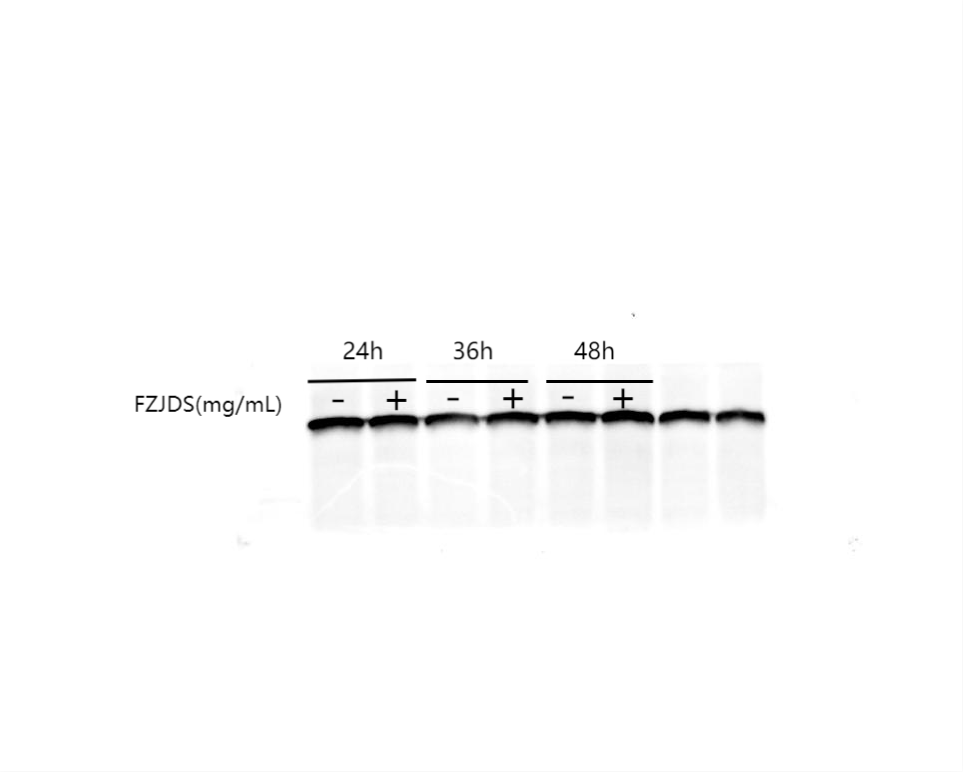

Supplement: S1 Data — (ZIP) [file pone.0283728.s001.zip › WB╘¡═╝/N╡░░╫╩▒╝Σ╥└└╡╫Θ/a┬-Actin.tif]

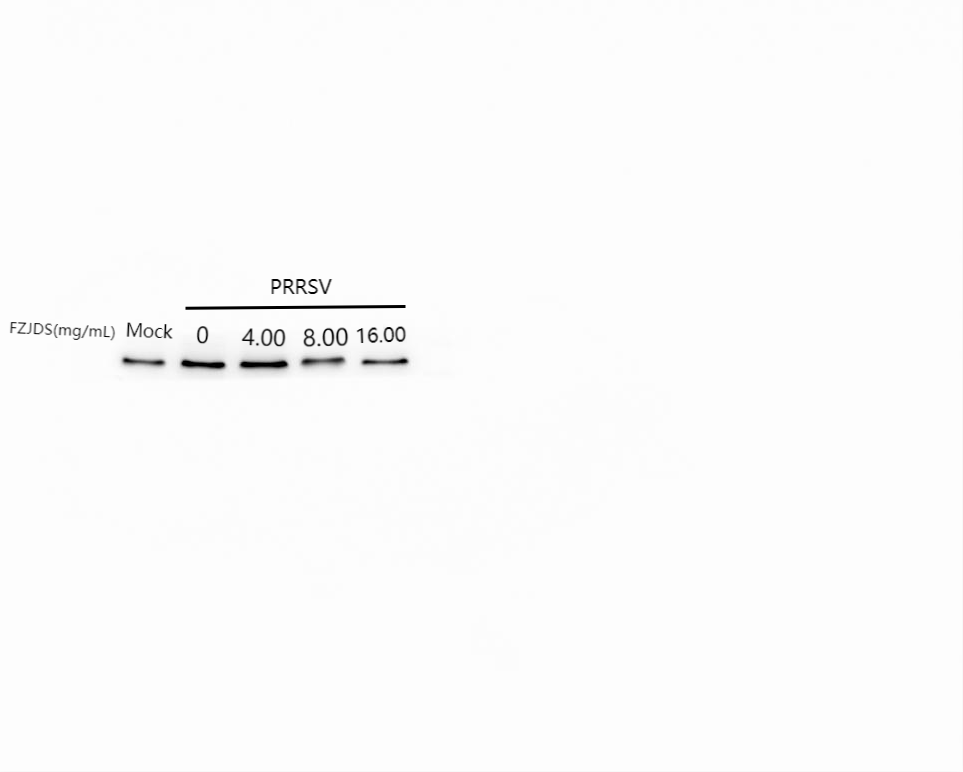

Supplement: S1 Data — (ZIP) [file pone.0283728.s001.zip › WB╘¡═╝/P-P65╥⌐╬∩╝┴┴┐╫Θ/P-p65.tif]

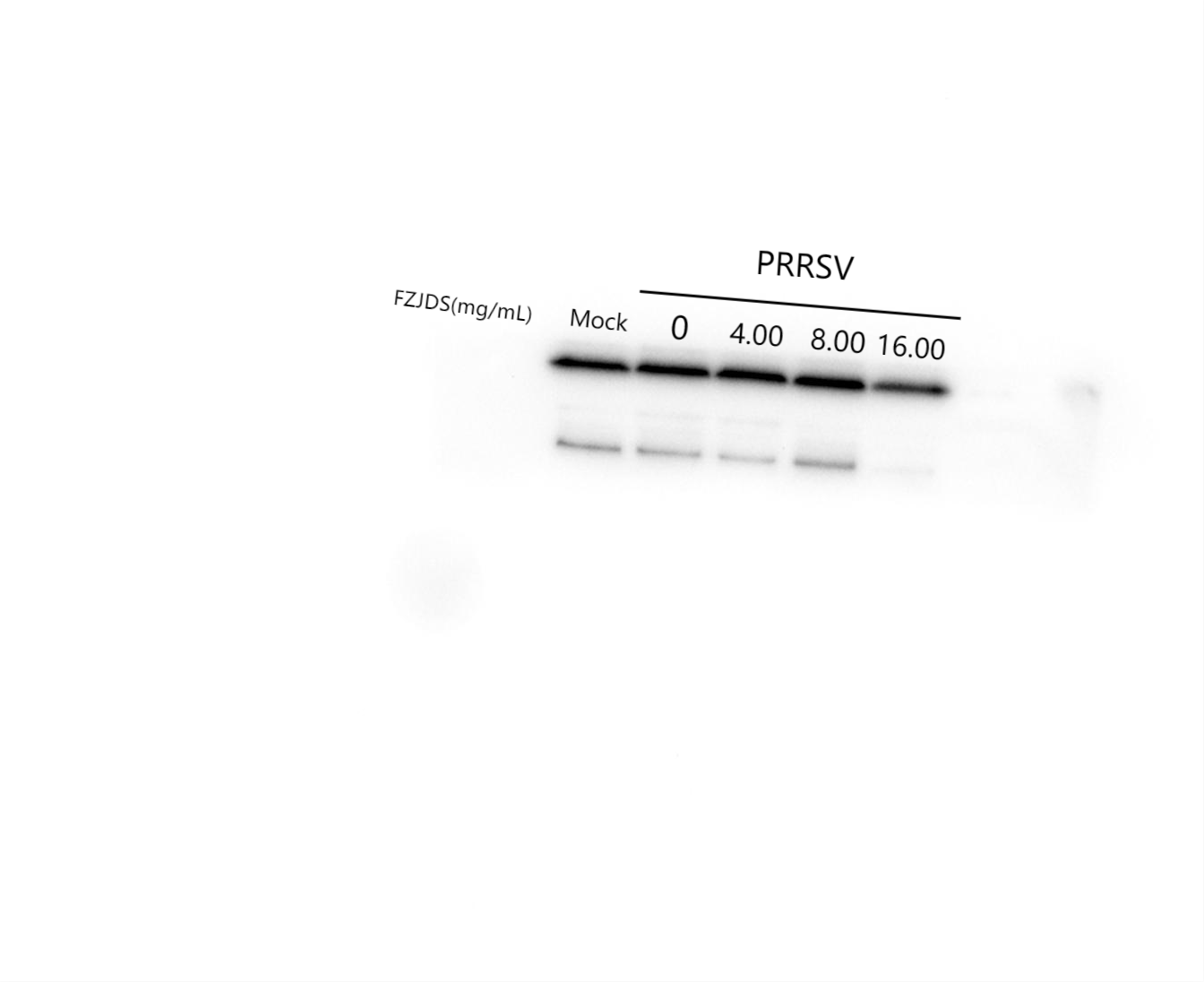

Supplement: S1 Data — (ZIP) [file pone.0283728.s001.zip › WB╘¡═╝/P-P65╥⌐╬∩╝┴┴┐╫Θ/p65.tif]

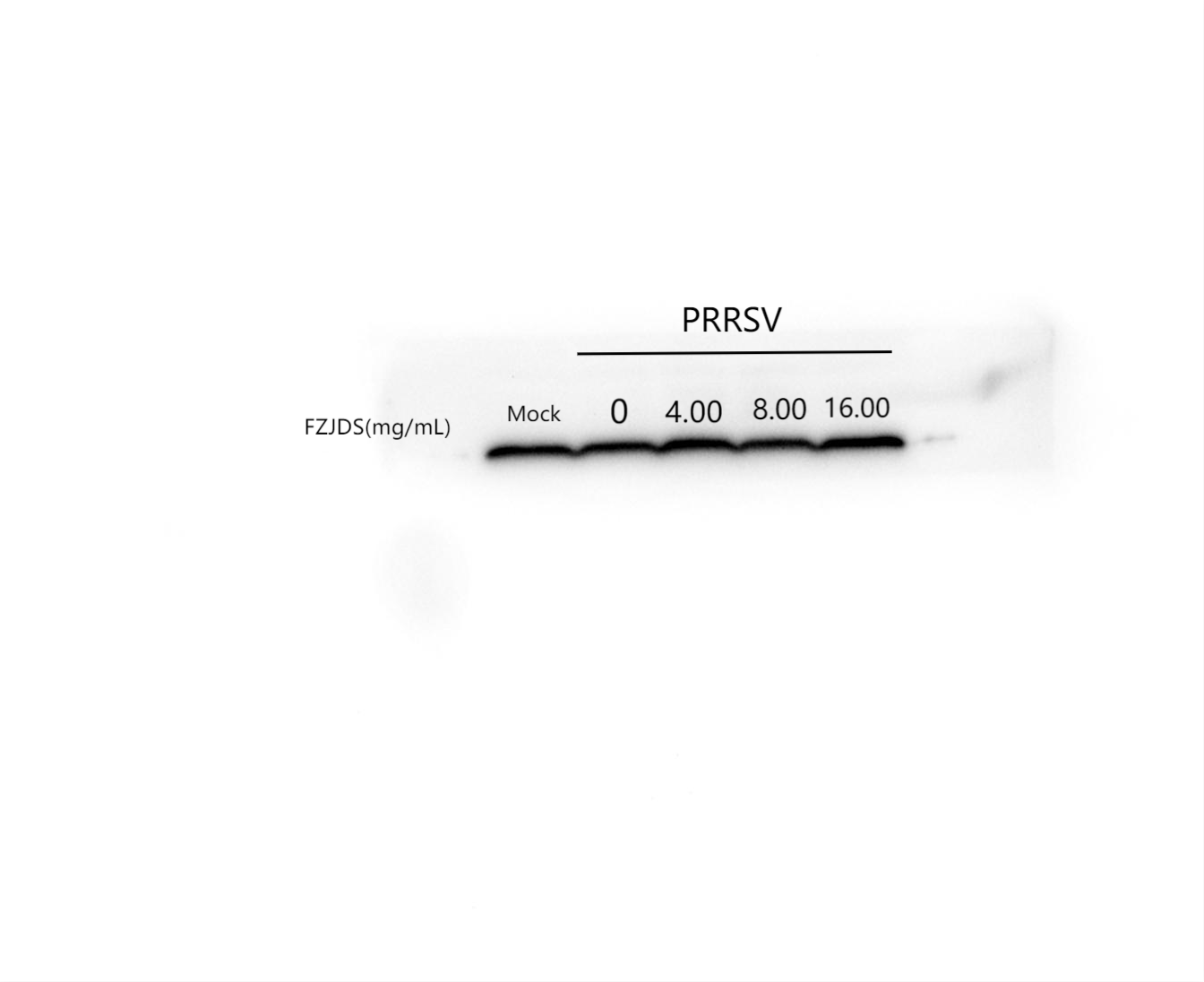

Supplement: S1 Data — (ZIP) [file pone.0283728.s001.zip › WB╘¡═╝/P-P65╥⌐╬∩╝┴┴┐╫Θ/a┬-Actin.tif]

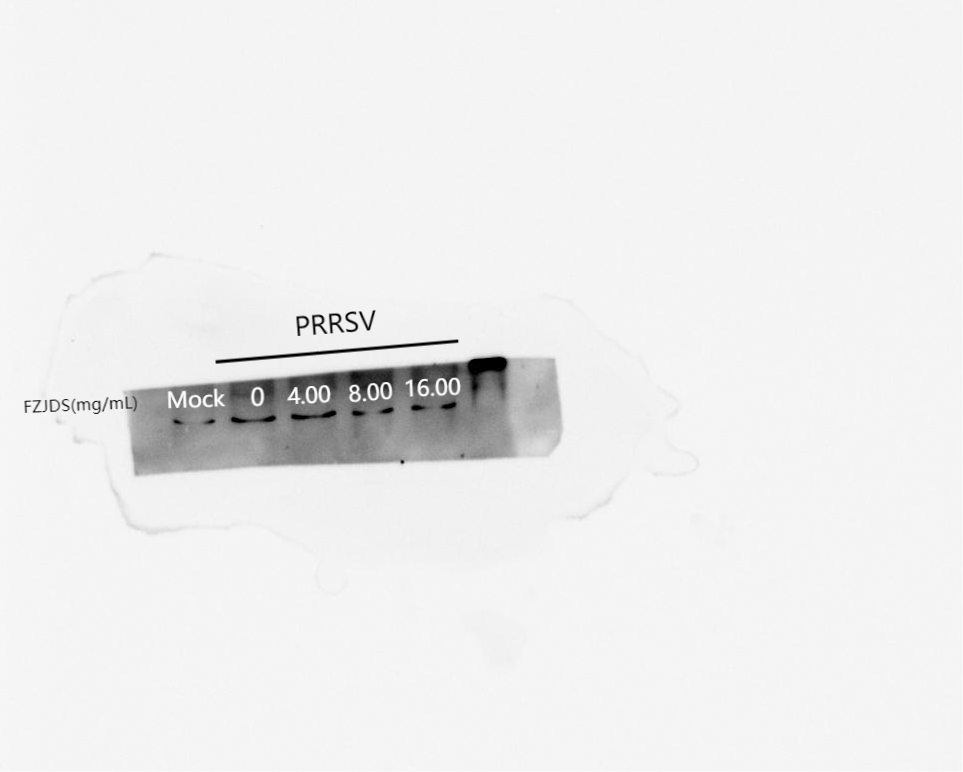

Supplement: S1 Data — (ZIP) [file pone.0283728.s001.zip › WB╘¡═╝/TLR4/TLR4.tif]

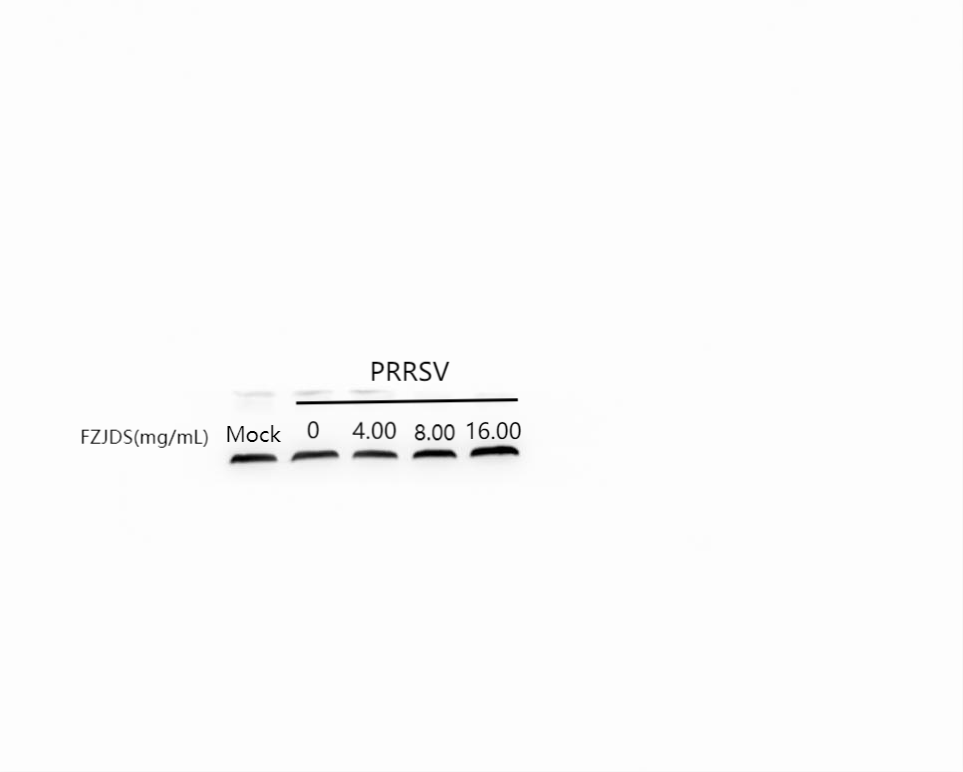

Supplement: S1 Data — (ZIP) [file pone.0283728.s001.zip › WB╘¡═╝/TLR4/a┬-Actin.tif]

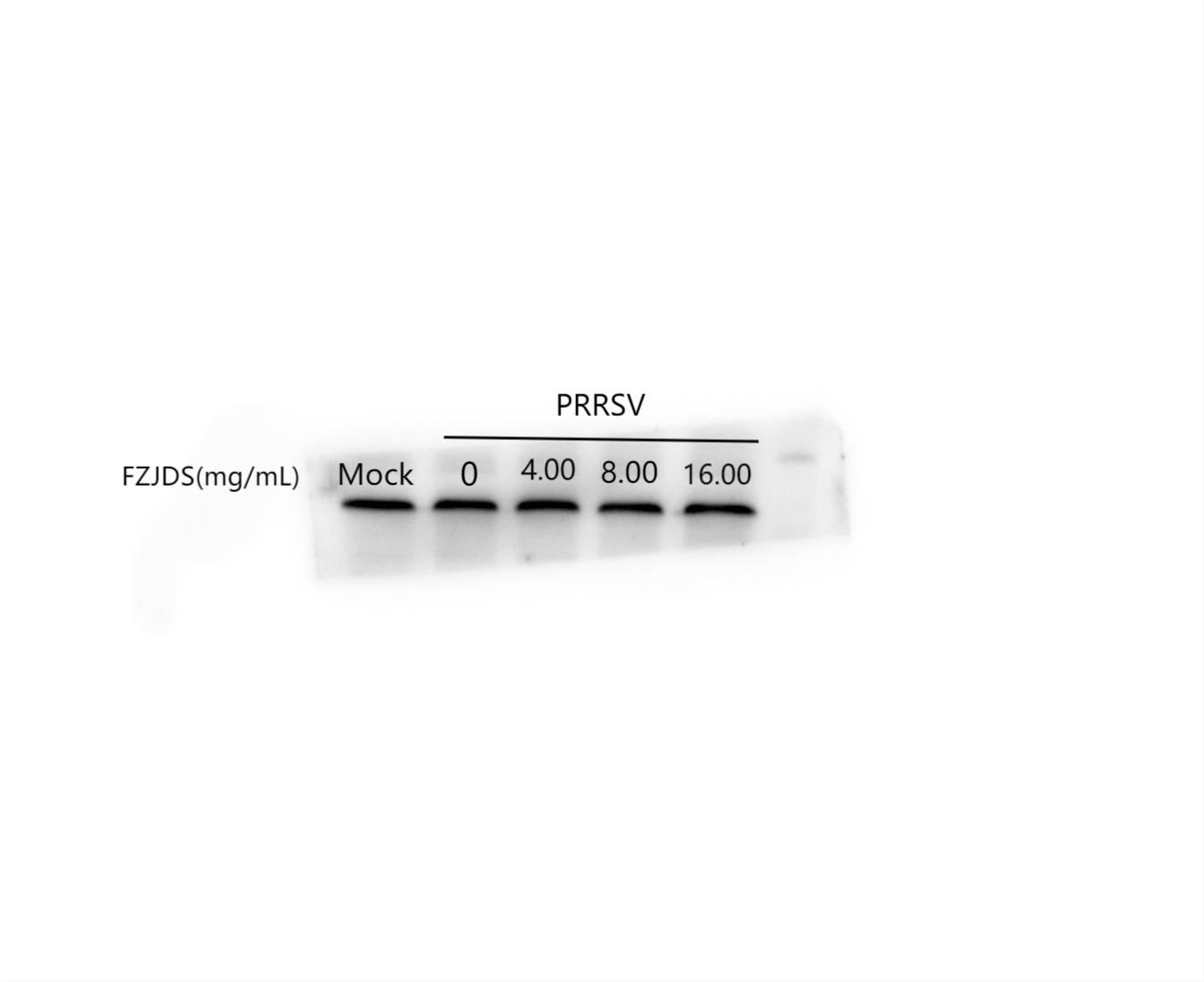

Supplement: S1 Data — (ZIP) [file pone.0283728.s001.zip › WB╘¡═╝/jnk╥⌐╬∩╝┴┴┐╫Θ/JNK a┬-Actin.tif]

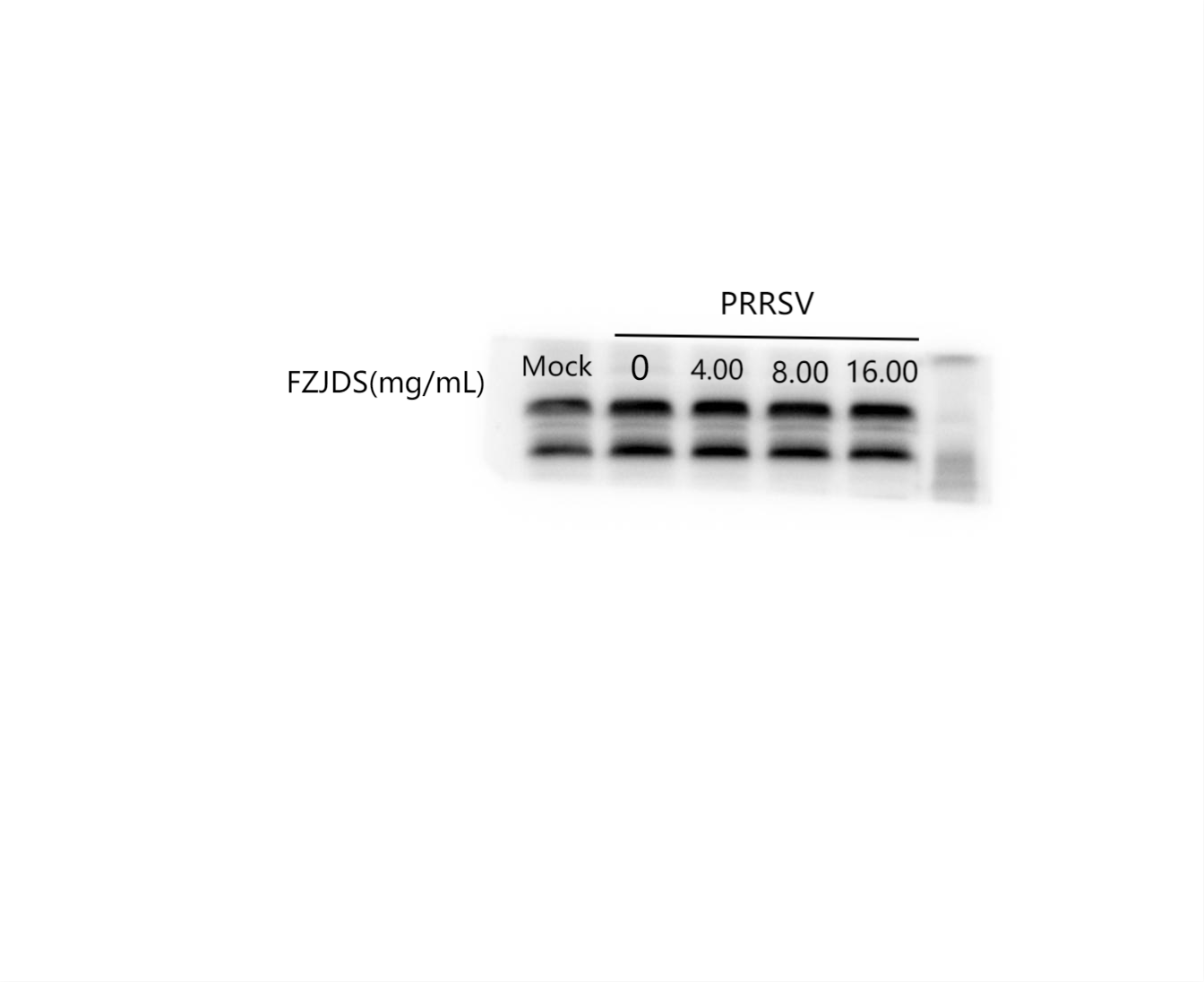

Supplement: S1 Data — (ZIP) [file pone.0283728.s001.zip › WB╘¡═╝/jnk╥⌐╬∩╝┴┴┐╫Θ/JNK.tif]

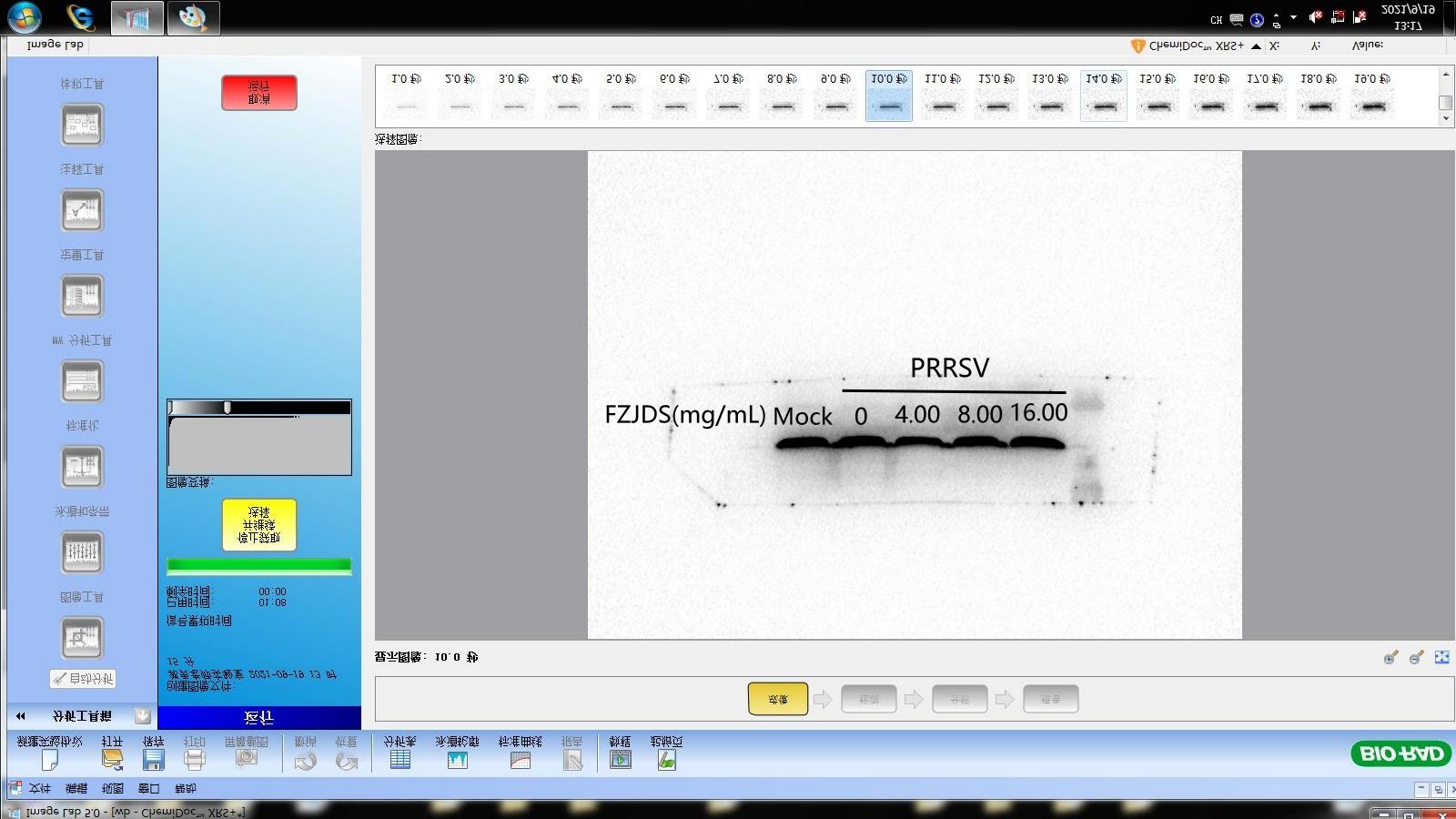

Supplement: S1 Data — (ZIP) [file pone.0283728.s001.zip › WB╘¡═╝/jnk╥⌐╬∩╝┴┴┐╫Θ/P-JNK a┬-Actin.jpg]

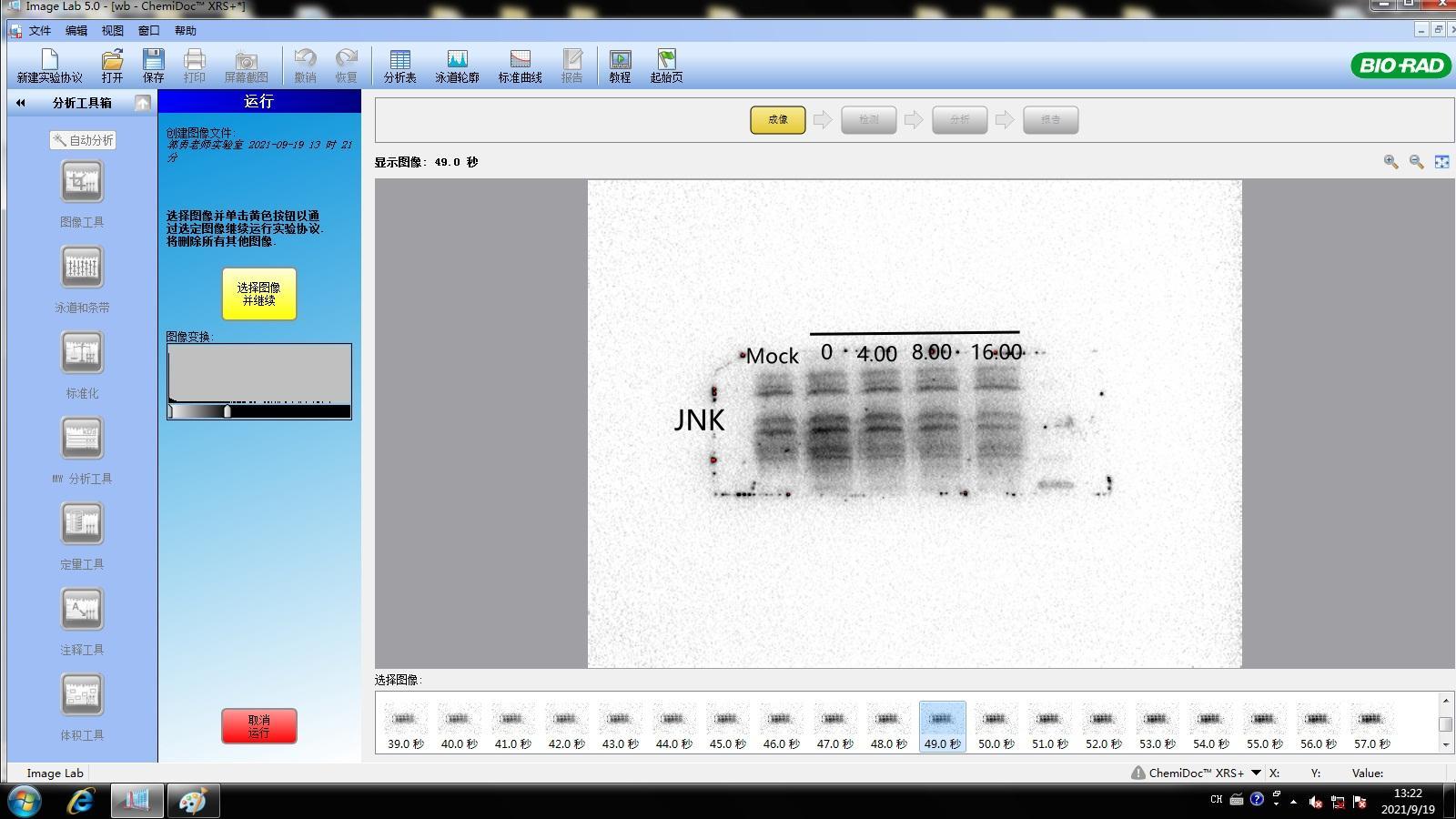

Supplement: S1 Data — (ZIP) [file pone.0283728.s001.zip › WB╘¡═╝/jnk╥⌐╬∩╝┴┴┐╫Θ/P-JNK.jpg]

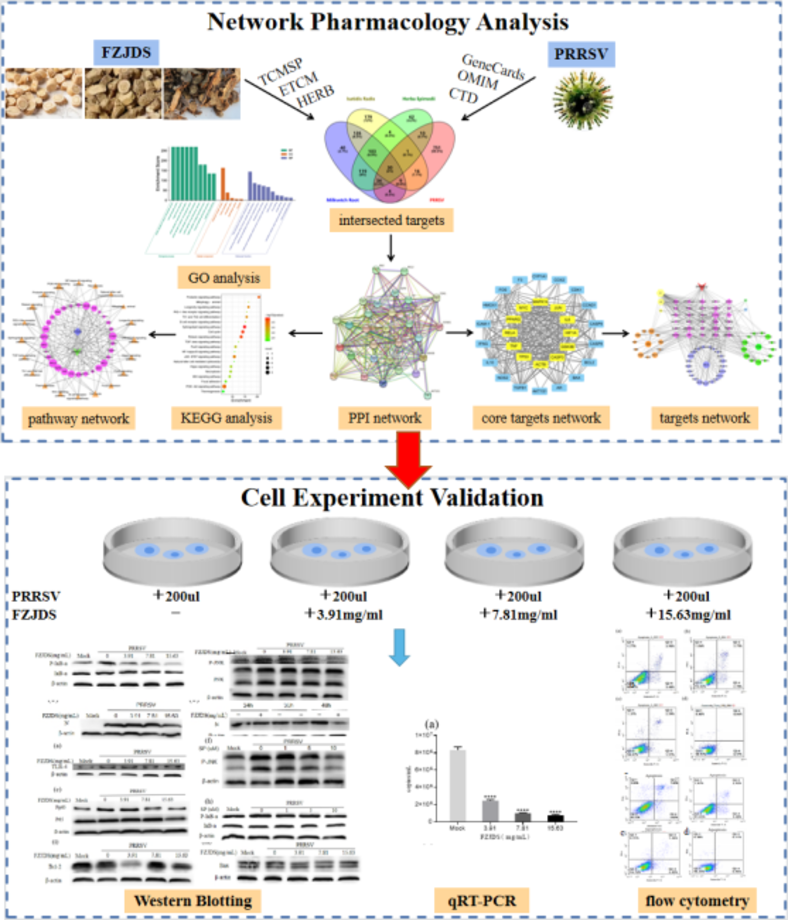

Supplement: S1 Graphical abstract — (TIF) [file pone.0283728.s002.tif]
