# Supplementary material for: Fuzhengjiedu San inhibits porcine reproductive and respiratory syndrome virus by activating the PI3K/AKT pathway
Source: PLoS One. 2024 May 6;19(5):e0283728. doi: 10.1371/journal.pone.0283728 (PMC11073700; doi:10.1371/journal.pone.0283728)
Supplement: Attachment — Submitted filename: Response to Reviewers-PLOS ONE.docx [file pone.0283728.s003.docx]

Dear Editors and Reviewers,

We highly appreciate the detailed and valuable comments of the reviewers on our manuscript entitled “Fuzhengjiedu San inhibits porcine reproductive and respiratory syndrome virus by activating the PI3K/AKT pathway” (ID: PONE-D-22-18907). As below, on behalf of my co-authors, I would like to clarify all the points raised by the reviewers. And we hope the reviewers and the editors will be satisfied with our responses to the comments and the revisions for the original manuscript.

The main corrections in the paper and the responds to the reviewer’s comments are as flowing:

Responds to the reviewer’s comments:

Reviewer #1:

1. Although network pharmacology provides important information about molecular interaction, it is difficult to make a comparative analysis between network pharmacology research and cytological research because of the huge difference between kidney cells （MARC‐ 145 cells derived from African green monkey kidney）selected and immune cells mainly from antiviral infection in cytological experiments.

Ans: Thank you for your comment. In the present study, we selected *sus scrofa* species during the network pharmacologic analysis, besides previous studies have been widely elucidated that the mechanism of PRRSV infection on swine by the MARC‐145 cell line. In a study reported from Xun Wang *et al* found that tea polyphenol effectively inhibited PRRSV infection in Marc-145 cells by suppressing the stages of viral attachment, internalization, replication and release (Xun Wang *et al.* 2021). In line with previous publications showed that whole-genome transcriptome analysis of PRRSV-infected Marc-145 cells, which provides insight into the dynamics of host gene regulation during PRRSV infection ([Ying Wei](https://pubmed.ncbi.nlm.nih.gov/?term=Wei%20Y%5bAuthor%5d) *et al.* 2018), as well as proved that PRRSV induced micropinocytosis via TIM-1 in MARC-145 cells ([Xin Wei](https://pubmed.ncbi.nlm.nih.gov/?size=200&term=Wei+X&cauthor_id=32522856) *et al.* 2020). Moreover, a proteomic study revealed that PRRSV virions incorporate a variety of simian proteins after replication in MARC-145 cells, which raises the intriguing possibility that cross-neutralization of PRRSV strains may, in particular, be mediated by antibodies recognizing simian antigen ([David Goldeck](https://pubmed.ncbi.nlm.nih.gov/?size=200&term=Goldeck+D&cauthor_id=30972067) *et al.* 2019). Previous findings suggest that the role of the plasma membrane cholesterol in PRRSV infection of MARC-145 cells was investigated by [Ying Sun](https://pubmed.ncbi.nlm.nih.gov/?size=200&term=Sun+Y&cauthor_id=22173307) *et al* (2011). While knockdown of DHX36 and its adaptor myeloid differentiation primary response gene 88 (MyD88) by small-interfering RNA in MARC-145 cells significantly reduced NF-kB activation and pro-inflammatory cytokine expression after PRRSV infection ([Huiyuan Jing](https://pubmed.ncbi.nlm.nih.gov/?size=200&term=Jing+H&cauthor_id=29123520) *et al.* 2017). Therefore, we used MARC‐145 cells in the present study for the cytological research validation.

We all totally agree with immune cells used to clarify the PRRSV infection mechanisms, rather than only either the MARC-145 cells or the immune effects. In the further research ,we'll both use immune cells for the mechanism study. Thanks for your suggestions again.

[1] Wang X, Dong W, Zhang X, Zhu Z, Chen Y, Liu X, Guo C. Antiviral Mechanism of Tea Polyphenols against Porcine Reproductive and Respiratory Syndrome Virus. Pathogens. 2021 Feb 13;10(2):202. doi: 10.3390/pathogens10020202. PMID: 33668502; PMCID: PMC7917843.

[2] Wei Y, Li J, Zhang Y, Xue C, Cao Y. Tandem 3' UTR Patterns and Gene Expression Profiles of Marc-145 Cells During PRRSV Infection. Virol Sin. 2018 Aug;33(4):335-344. doi: 10.1007/s12250-018-0045-y. Epub 2018 Aug 1. PMID: 30069823; PMCID: PMC6178098.

[3] Wei X, Li R, Qiao S, Chen XX, Xing G, Zhang G. Porcine Reproductive and Respiratory Syndrome Virus Utilizes Viral Apoptotic Mimicry as an Alternative Pathway To Infect Host Cells. J Virol. 2020 Aug 17;94(17):e00709-20. doi: 10.1128/JVI.00709-20. PMID: 32522856; PMCID: PMC7431799.

[4] Goldeck D, Perry DM, Hayes JWP, Johnson LPM, Young JE, Roychoudhury P, McLuskey EL, Moffat K, Bakker AQ, Kwakkenbos MJ, Frossard JP, Rowland RRR, Murtaugh MP, Graham SP. Establishment of Systems to Enable Isolation of Porcine Monoclonal Antibodies Broadly Neutralizing the Porcine Reproductive and Respiratory Syndrome Virus. Front Immunol. 2019 Mar 27;10:572. doi: 10.3389/fimmu.2019.00572. PMID: 30972067; PMCID: PMC6445960.

[5] Sun Y, Xiao S, Wang D, Luo R, Li B, Chen H, Fang L. Cellular membrane cholesterol is required for porcine reproductive and respiratory syndrome virus entry and release in MARC-145 cells. Sci China Life Sci. 2011 Nov;54(11):1011-8. doi: 10.1007/s11427-011-4236-0. Epub 2011 Dec 16. PMID: 22173307; PMCID: PMC7088586.

[6] Jing H, Zhou Y, Fang L, Ding Z, Wang D, Ke W, Chen H, Xiao S. DExD/H-Box Helicase 36 Signaling via Myeloid Differentiation Primary Response Gene 88 Contributes to NF-κB Activation to Type 2 Porcine Reproductive and Respiratory Syndrome Virus Infection. Front Immunol. 2017 Oct 23;8:1365. doi: 10.3389/fimmu.2017.01365. PMID: 29123520; PMCID: PMC5662876.

2. The initial concentration of the drug was 125 mg, because the use of multiple dilution resulted in the subsequent dose showing two decimal places（3.91，7.81mg/L，I do not think 3.91 is better than 4.0 here.）. This accuracy was not helpful for the analysis and understanding of its pharmacological effect, and the authors should reduce the number of decimal places. It is recommended to choose an initial dose of 128 mg, so that all dilutions are rounded and the numbers are tidy. Research is a very personal job and requires neat habits.

Ans: Thank you for your suggestion. It has been revised.

3. In order to determine the antiviral effect of the drug, an antiviral drug should also be used as a positive control, which is relatively easy to achieve for cell studies and can be more clearly illustrated. (3.5, Figure 6)

Ans: Thank you for your suggestion. Previous study by Liu Lan *et al.* (2016), Ma Xia *et al.*(2015) has proved that FZJDS plays an important role in anti-infectious Bursal Disease Virus(IBDV). Moreover, Guangwen Dai *et al.* (2017) described that FZJDS has a certain cure rate in pigs with clinical infection of PRRSV, Porcine circovirus type 2 (PCV2) and Pseudorabies virus (PRV). Therefore, in the present study we only test the different concentration of FZJDS effect on PRRSV infection cell model, which would like to select the best and safest concentration by the cell viability. In particular, in previous studies it has been confirmed by Zhengtu Li et al. (2017) found that the polysaccharide extract from *Radix isatidis* root can be used as an adjunct to antiviral therapy for the IAV infection and LH-C might be an option for treating influenza virus infection([Yuewen Ding](https://pubmed.ncbi.nlm.nih.gov/?term=Ding Y[Author]), 2017) without positive control. Therefore, no positive control is used in this study. We sincerely grateful all the reviewers' comment on the positive control group in the experiment design and will adding the positive controls in the following studies.

[1] Liu Lan, Li Shanshan, Wang Yafang, Peng Jinshan, Guo Zhihong, Zhang Junru, He Cheng. Clinical Efficacy of Fuzheng Jiedu Granules on Chickens Infected by IBDV [J].Chinese Journal of Veterinary Drug. 2016,50(09):41-46.

[2] Ma Xia, Guo Zhenhuan, Liu Yonglu, Wang Changlin, Zhang Guozu. Effects Comparision of Fuzheng Jiedu San and its Super Micropowder on Anti-IBDV in Vitro[J]. China Animal Husbandry & Veterinary Medicine. 2015,42(04):1032-1037.DOI:10.16431/j.cnki.1671-7236.2015.04.40.

[3] Dai Guangwen, Chen Sulian, Wei Bangwei, Jiang Jinghua, Xie Lihua, Wu Yinghang, Wu Zhiqiang. Screening test of different compound Chinese medicine preparations for prevention and treatment of swine hyperfebrile disease[J]. Chinese Journal of Traditional Veterinary Science. 2017(06):4-5.

[4] Li Z, Li L, Zhou H, Zeng L, Chen T, Chen Q, Zhou B, Wang Y, Chen Q, Hu P, Yang Z. Radix isatidis Polysaccharides Inhibit Influenza a Virus and Influenza A Virus-Induced Inflammation via Suppression of Host TLR3 Signaling In Vitro. Molecules. 2017,122(1):116-118. doi: 10.3390/molecules22010116. PMID: 28085062; PMCID: PMC6155848.

[5] Ding Y, Zeng L, Li R, Chen Q, Zhou B, Chen Q, Cheng PL, Yutao W, Zheng J, Yang Z, Zhang F. The Chinese prescription lianhuaqingwen capsule exerts anti-influenza activity through the inhibition of viral propagation and impacts immune function. BMC Complement Altern Med. 2017,17(1):130-142. doi: 10.1186/s12906-017-1585-7. PMID: 28235408; PMCID: PMC5324200.

4. The effects of FZJDS on uninfected cells were studied, but these effects were not analyzed. These effects are also necessary to analyze drug effects, so as to avoid non-specific concomitant effects.(3.6)

Ans: Thank you for your comment. In the present study, we analyzed the effects of FZJDS on uninfected cells in the mock group by western blot. Furthermore, we revised the discussion part hoping it better to elucidate the non-specific concomitant effects. We will be happy to edit the text further on the feedback comments on this study.

Reviewer #2:

1. The antiviral effects of FZJDS were only tested in Marc-145 cell, which is originated from green monkey. The natural target cell of PRRSV is PAM, the authors should use PAM to perform assays to demonstrate the anti-PRRSV activity of FZJDS.

Ans: Thank you for your comment. In the present study, we selected *sus scrofa* species during the network pharmacologic analysis, besides previous studies have been widely elucidated that the mechanism of PRRSV infection on swine by the MARC‐145 cell line. In a study reported from Xun Wang *et al* found that tea polyphenol effectively inhibited PRRSV infection in Marc-145 cells by suppressing the stages of viral attachment, internalization, replication and release (Xun Wang *et al.* 2021). In line with previous publications showed that whole-genome transcriptome analysis of PRRSV-infected Marc-145 cells, which provides insight into the dynamics of host gene regulation during PRRSV infection ([Ying Wei](https://pubmed.ncbi.nlm.nih.gov/?term=Wei%20Y%5bAuthor%5d) *et al.* 2018), as well as proved that PRRSV induced macropinocytosis via TIM-1 in MARC-145 cells ([Xin Wei](https://pubmed.ncbi.nlm.nih.gov/?size=200&term=Wei+X&cauthor_id=32522856) *et al.* 2020). Moreover, a proteomic study revealed that PRRSV virions incorporate a variety of simian proteins after replication in MARC-145 cells, which raises the intriguing possibility that cross-neutralization of PRRSV strains may, in part, be mediated by antibodies recognizing simian antigen ([David Goldeck](https://pubmed.ncbi.nlm.nih.gov/?size=200&term=Goldeck+D&cauthor_id=30972067) *et al.* 2019). Previous findings suggest that the role of the plasma membrane cholesterol in PRRSV infection of MARC-145 cells was investigated by [Ying Sun](https://pubmed.ncbi.nlm.nih.gov/?size=200&term=Sun+Y&cauthor_id=22173307) *et al* (2011). While knockdown of DHX36 and its adaptor myeloid differentiation primary response gene 88 (MyD88) by small-interfering RNA in MARC-145 cells significantly reduced NF-kB activation and pro-inflammatory cytokine expression after PRRSV infection ([Huiyuan Jing](https://pubmed.ncbi.nlm.nih.gov/?size=200&term=Jing+H&cauthor_id=29123520) *et al.* 2017). Therefore, we used MARC‐145 cells in the present study for the cytological research validation.

We all totally agree with immune cells used to clarify the PRRSV infection mechanisms, rather than only either the MARC-145 cells or the immune effects. In the further research ,we'll both use immune cells for the mechanism. Thanks for your suggestions again.

[1] Wang X, Dong W, Zhang X, Zhu Z, Chen Y, Liu X, Guo C. Antiviral Mechanism of Tea Polyphenols against Porcine Reproductive and Respiratory Syndrome Virus. Pathogens. 2021 Feb 13;10(2):202. doi: 10.3390/pathogens10020202. PMID: 33668502; PMCID: PMC7917843.

[2] Wei Y, Li J, Zhang Y, Xue C, Cao Y. Tandem 3' UTR Patterns and Gene Expression Profiles of Marc-145 Cells During PRRSV Infection. Virol Sin. 2018 Aug;33(4):335-344. doi: 10.1007/s12250-018-0045-y. Epub 2018 Aug 1. PMID: 30069823; PMCID: PMC6178098.

[3] Wei X, Li R, Qiao S, Chen XX, Xing G, Zhang G. Porcine Reproductive and Respiratory Syndrome Virus Utilizes Viral Apoptotic Mimicry as an Alternative Pathway To Infect Host Cells. J Virol. 2020 Aug 17;94(17):e00709-20. doi: 10.1128/JVI.00709-20. PMID: 32522856; PMCID: PMC7431799.

[4] Goldeck D, Perry DM, Hayes JWP, Johnson LPM, Young JE, Roychoudhury P, McLuskey EL, Moffat K, Bakker AQ, Kwakkenbos MJ, Frossard JP, Rowland RRR, Murtaugh MP, Graham SP. Establishment of Systems to Enable Isolation of Porcine Monoclonal Antibodies Broadly Neutralizing the Porcine Reproductive and Respiratory Syndrome Virus. Front Immunol. 2019 Mar 27;10:572. doi: 10.3389/fimmu.2019.00572. PMID: 30972067; PMCID: PMC6445960.

[5] Sun Y, Xiao S, Wang D, Luo R, Li B, Chen H, Fang L. Cellular membrane cholesterol is required for porcine reproductive and respiratory syndrome virus entry and release in MARC-145 cells. Sci China Life Sci. 2011 Nov;54(11):1011-8. doi: 10.1007/s11427-011-4236-0. Epub 2011 Dec 16. PMID: 22173307; PMCID: PMC7088586.

[6] Jing H, Zhou Y, Fang L, Ding Z, Wang D, Ke W, Chen H, Xiao S. DExD/H-Box Helicase 36 Signaling via Myeloid Differentiation Primary Response Gene 88 Contributes to NF-κB Activation to Type 2 Porcine Reproductive and Respiratory Syndrome Virus Infection. Front Immunol. 2017 Oct 23;8:1365. doi: 10.3389/fimmu.2017.01365. PMID: 29123520; PMCID: PMC5662876.

2. The cytotoxicity of FZJDS on Marc-145 should be shown in graph rather than table. Further, the authors should calculate the CC50 of the drug.

Ans: Thank you for your suggestion. We calculate CC50 value and add some necessary information on cytotoxicity in this manuscript.

Supplementary Figure 1. Cytotoxicity of FZJDS on MARC-145 cells. (a) 24 hour FZJDS treatment group. (b) 36 hour FZJDS treatment group. (c) 48 hour FZJDS treatment group. (d) 72 hour FZJDS treatment group.

3. The authors also should calculate the IC50, which is indispensable for antiviral drug research.

Ans: Thank you for your suggestion. The IC50 value has been added.

4. In Figure 6C and E, the inhibitory effect of FZJDS on PRRSV replication is very weak, indicating that the drug may not be used for PRRSV control.

Ans: Thank you for your comment. We double check and analysis the results of N protein by Image J software, which is a marker response to PRRSV infection. When Marc-145 cells added without or with FZJDS as the negative and positive control group, compared with only PRRSV positive control group, the content of N protein was significantly increased.

5. The level of p-p65 in mock treatment is very high and is almost comparable to that of PRRSV infection groups. Commonly the p-p65 level in mock group is very low or cannot be detectable. This phenomenon is also observed in the level of p-JNK and p-IkBalpha. The data are questionable.

Ans: We apologize for the mistake of phosphorylated proteins band in mock group intensity analysis in the manuscript. We re-analyzed on this results carefully, hoping it is better for understanding.

6. The data of bioinformatics shown in Figures 1-5 are too complex and difficult to follow, which makes the logic of the paper not clear.

Ans: Thank you for your comment. According to the analytical steps of network pharmacology, we list all the necessary results in line with the titles of each sections. Consistent with previous studies suggested that network pharmacology bioinformatics results in our study ([Wenjun Zhou](https://pubmed.ncbi.nlm.nih.gov/?size=200&term=Zhou+W&cauthor_id=34649337) *et al.* 2021,[Haoran Guo](https://pubmed.ncbi.nlm.nih.gov/?size=200&term=Guo+H&cauthor_id=34603597) *et al.* 2021,[Huahe Zhu](https://pubmed.ncbi.nlm.nih.gov/?size=200&term=Zhu+H&cauthor_id=34183011) *et al.* 2021). All the study demonstrate that network pharmacology is an effective tool for the discovery of natural compounds with specific properties and determination of their possible mechanisms. We will be happy to edit the text on the detailed comments on the network pharmacology results in this study.

[1] Zhou W, Zhu Z, Xiao X, Li C, Zhang L, Dang Y, Ge G, Ji G, Zhu M, Xu H. Jiangzhi Granule attenuates non-alcoholic steatohepatitis by suppressing TNF/NFκB signaling pathway-a study based on network pharmacology. Biomed Pharmacother. 2021 Nov;143:112181. doi: 10.1016/j.biopha.2021.112181. Epub 2021 Oct 4. PMID: 34649337.

[2] Guo H, Zeng H, Fu C, Huang J, Lu J, Hu Y, Zhou Y, Luo L, Zhang Y, Zhang L, Chen J, Zeng Q. Identification of Sitogluside as a Potential Skin-Pigmentation-Reducing Agent through Network Pharmacology. Oxid Med Cell Longev. 2021 Sep 23;2021:4883398. doi: 10.1155/2021/4883398. PMID: 34603597; PMCID: PMC8483913.

[3] Zhu H, Wang S, Shan C, Li X, Tan B, Chen Q, Yang Y, Yu H, Yang A. Mechanism of protective effect of xuan-bai-cheng-qi decoction on LPS-induced acute lung injury based on an integrated network pharmacology and RNA-sequencing approach. Respir Res. 2021 Jun 28;22(1):188. doi: 10.1186/s12931-021-01781-1. PMID: 34183011; PMCID: PMC8237774.
