# Supplementary material for: Fuzhengjiedu San inhibits porcine reproductive and respiratory syndrome virus by activating the PI3K/AKT pathway
Source: PLoS One. 2024 May 6;19(5):e0283728. doi: 10.1371/journal.pone.0283728 (PMC11073700; doi:10.1371/journal.pone.0283728)
Supplement: Attachment — Submitted filename: Response to Reviewers-PLOS ONE.docx [file pone.0283728.s004.docx]

Dear editor and reviewer,

We greatly appreciate the reviewer’s detailed and valuable comments on our manuscript entitled “Fuzhengjiedu San inhibits porcine reproductive and respiratory syndrome virus by activating the PI3K/AKT pathway” (ID: PONE-D-22-18907). On behalf of my coauthors, I would like to clarify our responses below to all the points raised by the reviewers. We hope that the reviewers and the editor will be satisfied with the changes and additions made to the original manuscript in the revised file. Their critiques have made the paper better and we thank you again for your kind consideration.

The responses to the reviewer’s comments and details of the corrections follow:

Reviewer #1

1. The key evidence to study the antiviral effect of drugs is the influence of drugs on the proliferation level of the virus. After cells are infected with the virus, PCR or cell staining can be used for quantitative analysis of the virus, so as to preliminarily determine the antiviral effect of drugs. This study is only from the perspective of the cell effect of the virus, lacking the most critical antiviral effect. This does not meet the basic requirements of a complete scientific story.

Ans: Thank you for your comment. We strongly agree with your suggestion about quantitating viral proliferation to make a more complete story and this is planned for the follow-up study. In the present study, we followed the pattern of previously published work and used RT-qPCR and western blotting to determine the inhibitory effects of FZJDS on PRRSV, in line with other researchers’ methods for testing the antiviral effect. For example, Ruansit, et al. [1] evaluated the in vitro antiviral activities and the ex vivo immunomodulatory effects of *Houttuynia cordata* Thunb through RT-qPCR, and the in vitro MTT assay was used by Jiang, et al. [2] to measure the effect of *Isatis* root polysaccharide on PRRSV infection. To assess the inhibitory activity of another key component of FZJDS, RT-qPCR was employed by Su Ke et al. [3] to determine the effect of radix isatidis on the replication pathway and proliferation of PRRSV. Our current study followed previous experimental methods, but we do plan to perform cell staining in a subsequent analysis of the antiviral effect and determine actual viral titers. Thanks again for this valuable suggestion.

References:

[1] Ruansit W, Charerntantanakul W. Oral Supplementation of Houttuynia cordata Extract Reduces Viremia in PRRSV-1 Modified-Live Virus-Vaccinated Pigs in Response to the HP-PRRSV-2 Challenge. Front Immunol. 2022 Jul 18;13:929338. doi: 10.3389/fimmu.2022.929338.

[2] Jiang D, Zhang L, Zhu G, Zhang P, Wu X, Yao X, Luo Y, Yang Z, Ren M, Wang X, Chen S, Wang Y. The Antiviral Effect of Isatis Root Polysaccharide against NADC30-like PRRSV by Transcriptome and Proteome Analysis. Int J Mol Sci. 2022 Mar 28;23(7):3688. doi: 10.3390/ijms23073688.

[3] DOI: 10.16303/j.cnki.1005-4545.2022.12.24.

2. The antiviral effect of cells should not be the primary function of the (PI3K)/Akt pathway, which is a key pathway for cell survival and, of course, one of the phenomena studied in this manuscript, the reduction of apoptosis effect. There are also more downstream molecules involved in the process of viral infection. For example, the mTOR gene can reduce the cell vitality of influenza virus infection, and the downregulation of its gene can enhance cell apoptosis. The downregulation of the gene expression can also reduce the virus titer in cells, indicating that mTOR can maintain the cell vitality of influenza virus infection and negatively regulate the apoptosis of influenza virus infection cells. The effective replication of influenza virus is ensured by maintaining cell viability and reducing cell apoptosis. The down-regulation of GSK-3β gene expression can reduce and increase the cell viability of influenza virus infection.

The apoptosis induced by strong influenza virus decreased the titer of influenza virus in cells, suggesting that GSK-3β had a negative regulatory effect on apoptosis and promoted the replication of influenza virus by maintaining the vitality of host cells. All of these molecules are downstream of the (PI3K)/Akt pathway and are conducive to viral infection, but generally do not have antiviral effects. For example, studies have shown that Shufeng Xuanfei recipe and Jiebiao Qingli recipe can significantly down-regulate the mRNA transcription levels of AKT, Fas, FasL and Caspase-9 in the signaling pathway, and reduce the protein expression levels of AKT, Fas and FasL, thus playing an anti-influenza role.

Ans: Thank you for taking the time to make this detailed comment. Your insights and information were very helpful for revising and improving our paper. In this study, the key reason why we selected the PI3K/AKT pathway is that it was the predominant result indicated by KEGG enrichment analysis as well as involved the core proteins most associated with PRRSV infection. Based on references from pharmacological network searching, the PI3K/AKT pathway was most often selected to verify antiviral activity by western immunoblotting and flow cytometry. A recent genome-wide mRNA and long non-coding RNA (lncRNA) search and analysis in PRRSV-infected PTR2 showed that PRRSV infection could inhibit the PI3K/AKT/mTOR pathway and trigger apoptosis [1]. Another study determined the phosphorylation status of Akt during infection of MARC-145 cells with the highly pathogenic PRRSV strain, HuN4, and provided new evidence of a novel role for the PI3K/Akt pathway in PRRSV infection [2]. Thus, we have modified the discussion section to include Bax, Bcl-2, TLR4 and p53, which have been identified as the core target proteins of FZJDS, in line with a previous report that protocatechuic acid suppressed H1N1-induced TLR4/NF-κB activation [3]. Previous studies revealed that bovine ephemeral fever virus disrupted Beclin 1 and Bcl-2 interaction by JNK-mediated Bcl-2 phosphorylation, thereby activating autophagy [4], which proves that JNK and Bcl-2 are among the chief proteins affecting PRRSV proliferation. Activated JNK translocates to the mitochondria where it phosphorylates Bcl-2/Bcl-xL and antagonizes the anti-apoptotic activity of Bcl-2/Bcl-xL. We incorporated these results into the present study discussion in hopes of clarifying the antiviral mechanism according to your comments and making the paper better. We would be happy to take any further feedback.

References:

[1] Zhang X, Liu X, Peng J, Song S, Xu G, Yang N, Wu S, Wang L, Wang S, Zhang L, Liu Y, Liang P, Hong L, Xu Z, Song C. Genome-Wide mRNA and Long Non-Coding RNA Analysis of Porcine Trophoblast Cells Infected with Porcine Reproductive and Respiratory Syndrome Virus Associated with Reproductive Failure. Int J Mol Sci. 2023 Jan 4;24(2):919. doi: 10.3390/ijms24020919.

[2] Zhu L, Yang S, Tong W, Zhu J, Yu H, Zhou Y, Morrison RB, Tong G. Control of the PI3K/Akt pathway by porcine reproductive and respiratory syndrome virus. Arch Virol. 2013 Jun;158(6):1227-34. doi: 10.1007/s00705-013-1620-z.

[3] Qian Wang, Xiaojuan Ren, Jinhua Wu, Hongrong Li, Liu Yang, Yan Zhang, Xin Wang, Zhicun Li, Eur J Clin Microbiol Infect Dis. 2022 Jan 24;1-8. [https://doi.org/10.1007/s10096- 022-04401-y.](https://doi.org/10.1007/s10096-022-04401-y.)

[4] Ching-Yuan Cheng, Hsu‑Hung Tseng, Hung‑Chuan Chiu, Ching‑Dong Chang, Brent L. Nielsen, Vet Res (2019) 50:79, https://doi.org/10.1186/s13567-019-0697-0.

3. There are two types of cellular antiviral effects. One is the production of molecules that kill the virus, such as antibodies and interferon. Instead, the cell commits suicide, allowing the infected virus to disappear along with the cell. From this perspective, the signaling pathways that promote cell survival are not conducive to the antiviral purposes of multicellular organisms.

Has a network pharmacological approach been used to study the antiviral molecular basis of this drug, but the effect of this drug on the (PI3K)/Akt pathway has not been found? How does the author explain why similar research methods and objectives produce such different conclusions?

Ans: We greatly appreciate your insightful comments and suggestions. We have read the article (DOI:10.16466/j.issn1005-5509.2020.08.012.) and found that the main components of the Fuzheng Jiedu formulation used in that study were white *Rhizoma* *atrictyloides*, tangerine peel, parsnip, licorice, patchouli, Astragalus membranaceus, honeysuckle and forsythiae. However, the Fuzheng Jiedu San used by us is a combination of Isatis root, Astragalus membranaceus and Herba medii. Due to significant differences in drug compatibility as delineated by network pharmacological analysis, the components, targets and pathways of these formulations are totally different.

The drug compatibility of traditional Chinese medicine preparations may increase or decrease according to the disease, and there has been no network pharmacological antiviral study on a formulation with the same components as used in this study; however, there have been reports on related single drugs such as from *Abelmoschus manihot* that can inhibit IAV-induced lung inflammation [1]. A network pharmacology investigation of the anti-inflammatory and antiviral effects of *Isatis* *indigotica* identified lignans that might regulate the immune system by targeting IL-2 and the PI3K/Akt signaling pathway [2]. Radix isatidis was analyzed through network pharmacology technology and it was found that COVID-19 could be inhibited by targeting the signaling pathways involving receptors for advanced glycation end products, interleukin-17, tumor necrosis factor, sphingolipids, and p53 [3].

References:

[1] Gao Y, Liang Z, Lv N, Shan J, Zhou H, Zhang J, Shi L. Exploring the total flavones of Abelmoschus manihot against IAV-induced lung inflammation by network pharmacology. BMC Complement Med Ther. 2022 Feb 5;22(1):36. doi: 10.1186/s12906-022-03509-0.

[2] Deng J, Ma Y, He Y, Yang H, Chen Y, Wang L, Huang D, Qiu S, Tao X, Chen W. A Network Pharmacology-Based Investigation to the Pharmacodynamic Material Basis and Mechanisms of the Anti-Inflammatory and Anti-Viral Effect of Isatis indigotica. Drug Des Devel Ther. 2021 Jul 20;15:3193-3206. doi: 10.2147/DDDT.S316701.

[3] Yu B, Lin F, Ning H, Ling B. Network pharmacology study on the mechanism of the Chinese medicine Radix Isatidis (Banlangen) for COVID-19. Medicine (Baltimore). 2021 Aug 13;100(32):e26881. doi: 10.1097/MD.0000000000026881.

We appreciate the reviewer’s detailed and insightful comments and hope that the revised manuscript will meet with their approval. Thanks to all for your help.

Sincerely,

Jinjin Tong
